# Multiple Ehrlichia chaffeensis Genes Critical for Its Persistent Infection in a Vertebrate Host Are Identified by Random Mutagenesis Coupled with *In Vivo* Infection Assessment

**DOI:** 10.1128/IAI.00316-20

**Published:** 2020-09-18

**Authors:** Ying Wang, Arathy D. S. Nair, Andy Alhassan, Deborah C. Jaworski, Huitao Liu, Kathleen Trinkl, Paidashe Hove, Charan K. Ganta, Nicole Burkhardt, Ulrike G. Munderloh, Roman R. Ganta

**Affiliations:** a Center of Excellence for Vector-Borne Diseases (CEVBD), Department of Diagnostic Medicine/Pathobiology, College of Veterinary Medicine, Kansas State University, Manhattan, Kansas, USA; b Department of Pathobiology, School of Veterinary Medicine, St. George’s University, West Indies, Grenada; c Department of Entomology, University of Minnesota, St. Paul, Minnesota, USA; Washington State University

**Keywords:** *Ehrlichia chaffeensis*, mutagenesis, transposon, *in vivo* screening, tick-borne diseases, rickettsial, tick-borne pathogens

## Abstract

Ehrlichia chaffeensis, a tick-transmitted obligate intracellular rickettsial agent, causes human monocytic ehrlichiosis. In recent reports, we described substantial advances in developing random and targeted gene disruption methods to investigate the functions of E. chaffeensis genes. We reported earlier that the Himar1 transposon-based random mutagenesis is a valuable tool in defining *E. chaffeensis* genes critical for its persistent growth *in vivo* in reservoir and incidental hosts. The method also aided in extending studies focused on vaccine development and immunity. Here, we describe the generation and mapping of 55 new mutations. To define the critical nature of the bacterial genes, infection experiments were carried out in the canine host with pools of mutant organisms. Infection evaluation in the physiologically relevant host by molecular assays and by xenodiagnoses allowed the identification of many proteins critical for the pathogen’s persistent *in vivo* growth. Genes encoding proteins involved in biotin biosynthesis, protein synthesis and fatty acid biosynthesis, DNA repair, electron transfer, and a component of a multidrug resistance (MDR) efflux pump were concluded to be essential for the pathogen’s *in vivo* growth. Three known immunodominant membrane proteins, i.e., two 28-kDa outer membrane proteins (P28/OMP) and a 120-kDa surface protein, were also recognized as necessary for the pathogen’s obligate intracellular life cycle. The discovery of many *E. chaffeensis* proteins crucial for its continuous *in vivo* growth will serve as a major resource for investigations aimed at defining pathogenesis and developing novel therapeutics for this and related pathogens of the rickettsial family *Anaplasmataceae*.

## Abstract (precis)

Ehrlichia chaffeensis, a tick-transmitted obligate intracellular rickettsial agent, causes human monocytic ehrlichiosis. In recent reports, we described substantial advances in developing random and targeted gene disruption methods to investigate the functions of E. chaffeensis genes. We reported earlier that the Himar1 transposon-based random mutagenesis is a valuable tool in defining *E. chaffeensis* genes critical for its persistent growth *in vivo* in reservoir and incidental hosts.

## INTRODUCTION

During the past 3 decades, rickettsial diseases caused by *Anaplasmataceae* family pathogens in the genera *Ehrlichia* and *Anaplasma* have emerged as a growing public health concern (1–8), and they are now considered the second leading human tick-borne diseases in the United States and many parts of the world. These diseases include human monocytic and granulocytic ehrlichiosis caused by Ehrlichia chaffeensis and Ehrlichia ewingii, respectively, which are transmitted by Amblyomma americanum, and human granulocytic anaplasmosis resulting from infection with Anaplasma phagocytophilum, which is transmitted by *Ixodes* species ticks. Recently, another Ixodes scapularis-borne pathogen, Ehrlichia muris subsp. *eauclairensis*, has been recognized as the causative agent for another ehrlichial disease in people (7, 8). Despite the complex life cycle involving tick vectors and vertebrate hosts, the rickettsial pathogens evolved strategies to evade clearance by both vertebrate and acarine hosts.

Like human beings, dogs acquire E. chaffeensis from infected A. americanum ticks (9). The limited availability of genetic tools to study obligate intracellular rickettsiae of the genera *Ehrlichia*, *Anaplasma*, *Rickettsia*, and *Orientia* is a major constraint for investigations focused on defining the functions of genes contributing to bacterial pathogenesis (10). In this context, genetic factors associated with persistent infections are of particular interest (11–13). Lack of well-established mutagenesis methods for rickettsial agents has remained a major impediment for advancing research to understand the functions of many uncharacterized bacterial genes. Recently, we reported the development of random and targeted mutagenesis methods for *E. chaffeensis* (14). We described targeted mutagenesis resulting in disruption of gene function, as well as in restoration of the function of a mutated gene (15). We further reported that Himar1 transposase-based random mutagenesis is efficient in creating mutations in both protein-coding and noncoding regions of the pathogen (14). We demonstrated that Himar1 mutagenesis is a valuable tool in elucidating host-pathogen interactions and in developing attenuated mutant vaccines (16–18). Similarly, random mutagenesis is described for other members of the alphaproteobacterial order *Rickettsiales* (19–24).

In the current study, we generated a random mutagenesis library for *E. chaffeensis* and mapped 55 insertion mutations. The transposon insertion mutants were utilized for *in vivo* screening experiments in a physiologically relevant canine host infection model. The study aided in identifying many essential genes and genomic regions of *E. chaffeensis*.

## RESULTS

### Transposon mutagenesis library of *E. chaffeensis*.

**FIG 1:**
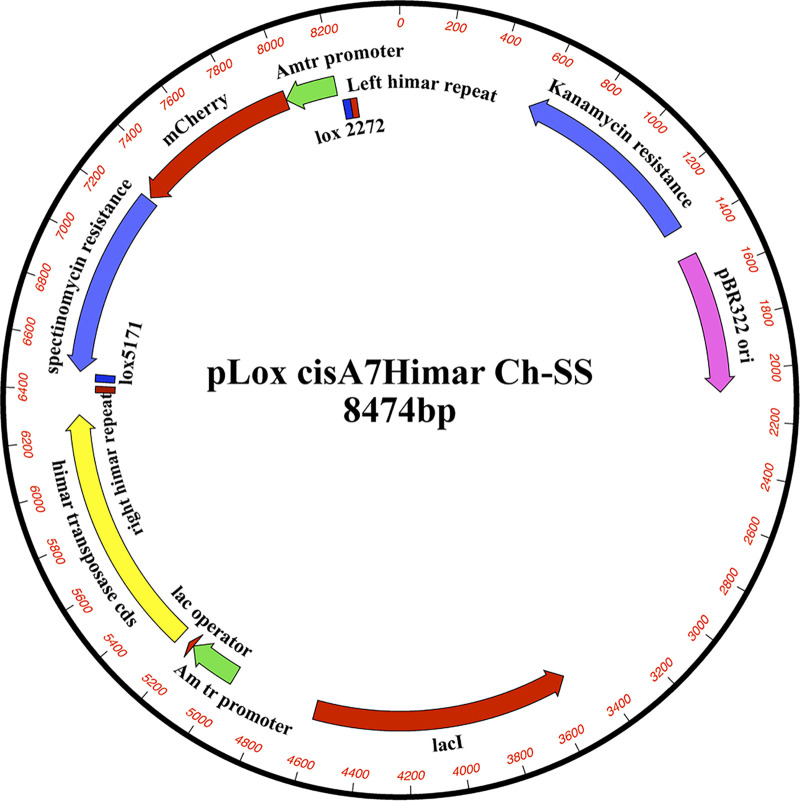
Plasmid map of pHimar1 A7 loxP used for the electroporation to generate a subset of mutants in *E. chaffeensis*.

**FIG 2:**
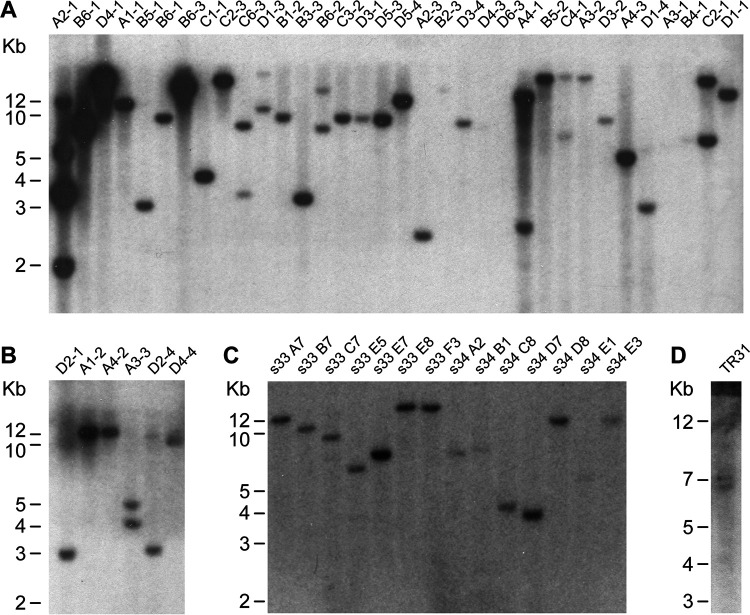
Southern blot analysis of *E. chaffeensis* Himar1 mutants. Genomic DNA from Himar1 transposon *E. chaffeensis* mutants was assessed by DNA blot analysis using a spectinomycin resistance gene (*aadA*) probe. Panels A to D represent four DNA blot analysis experimental data generated four independent times to locate all insertion mutations within *E. chaffeensis* genome. Genomic DNAs from the cultured mutants were digested with BglII restriction enzyme (mutants’ codes are identified at the top). Genomic locations for the DNA fragments (identified in Fig. S1) were established by sequence analysis.

We previously reported the application of Himar1 transposon mutagenesis in creating mutations spanning 9 genomic regions of *E. chaffeensis* (14). The mutants served as a resource in mapping genes critical for the pathogen and in studies focused on developing a live attenuated vaccine (16, 17). In the current study, we extended the transposon mutagenesis by performing several independent mutational experiments using the ISE6 tick cell line and with three different mutagenesis constructs; all three constructs had the *aadA* gene to confer resistance to spectinomycin/streptomycin, while two constructs contained an mCherry expression cassette and the third construct had a green fluorescent protein (GFP) expression cassette (25–28). One of the two mCherry-expressing constructs (pHimar1 A7 loxP plasmid) also included Cre-*loxP* flanking sequences; it was generated by insertion into mismatched *loxP* sites flanking the transposon segment in pCis mCherry-SS Himar A7 (Fig. 1). The mutagenesis experiments with all three plasmids aided in the identification of many insertion mutations, as judged from Southern blot analysis of genomic DNAs recovered from the mutant organisms (Fig. 2). The DNA blot analysis was performed using genomic DNAs of the mutants digested with BglII, as the insertion sequence lacked the recognition sequence for this restriction enzyme. All three mutagenesis constructs performed similarly in generating mutants, with few mutations identified after each experiment. The majority of the mutants were clonally pure, with the exception of a few having a mix of two or more mutants (Fig. 2).

### Mapping the genomic insertion sites.

**FIG 3:**
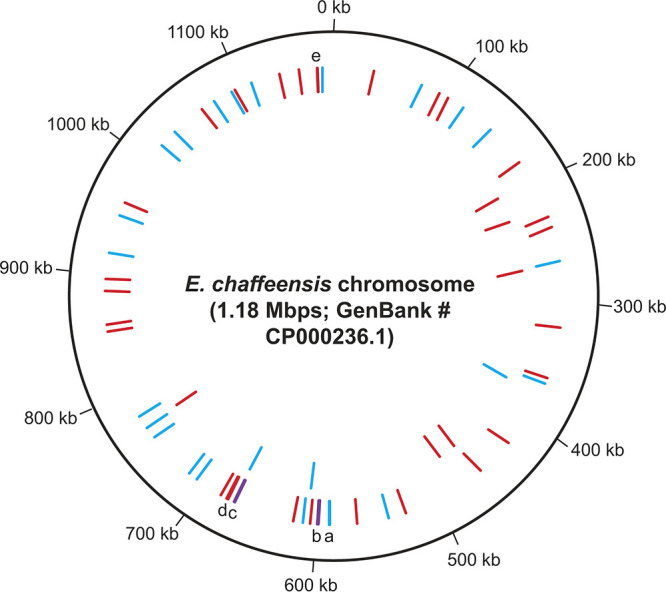
*E. chaffeensis* chromosomal map showing all 55 transposons insertion sites identified in the current study. The circular representation of *E. chaffeensis* chromosomal map was created using DNAplotter (79). The red lines indicate mutations within the coding regions of genes, and the blue lines refer to mutations within intergenic regions of genes. (The letters a, b, c, d, and e refer to the mutant lines where more than one insertion mutation is present at close proximities and they could not be separated in the image. Purple lines b and c are to represent the presence of intergenic and intragenic mutations at close vicinity.) The previously mapped 9 mutations (14) are also included (inner circle lines).

To establish the identity of the mutant insertion sites, genome-walking PCRs and sequencing analyses were performed using the genomic DNAs recovered from the mutant organisms as templates. We mapped 55 transposon insertion sites to the *E. chaffeensis* genome from the mutant library (Fig. 3 and Tables 1 and 2), while the identity of a few insertion mutations remains to be defined (see Fig. S1 in the supplemental material). Including the previously reported 9 transposon mutations (14), the total number of insertion mutations in *E. chaffeensis* genome is 64 (Fig. 3). The mutation sites were distributed randomly throughout the *E. chaffeensis* genome, although we did not identify mutations in some major genomic segments spanning regions of about 25 to 50 kbp (Fig. 3). Furthermore, there were about equal numbers of insertions found within the open reading frames (ORFs) of genes (31 mutants) (Table 1) and in the intergenic spaces (24 mutants) (Table 2).

**TABLE 1:** *E. chaffeensis* Himar1 transposon insertion mutants in ORFs (total, 31)

Insertion cassette	Mutant no.	Mutant code	Gene no.^*a*^	Gene product	Genomic insertion location^*a*^	Insertion orientation^*b*^	Insertion location in ORF/ORF length (bp)
*mCherry loxP*	1	A1-1	ECH_0113	Hypothetical protein	99608	+	924/2,382
	2	A3-2	ECH_0187	Hypothetical protein	176793	−	1154/1,692
	3	D4-1	ECH_0242	Hypothetical protein	226758	+	115/162
	4	s33 E5	ECH_0251	Hypothetical protein	236424	+	137/618
	5	D3-2	ECH_0368	Dioxygenase family protein	360362	+	243/675
	6	s34 C8	ECH_0445	Queuine tRNA-ribosyltransferase	423364	+	352/1,191
	7	C1-1	ECH_0475	Signal recognition particle protein	454669	−	1302/1,347
	8	s33 C7	ECH_0525	Hypothetical protein	525880	−	1037/2,001
	9	s34 E3	ECH_0592	Coproporphyrinogen III oxidase, aerobic, truncation	598900	−	83/147
	10	B5-1	ECH_0600	Hypothetical protein	606371	+	70/144
	11	D3-1	ECH_0614	Hypothetical protein	619640	−	334/696
	12	B1-2	ECH_0655	RNA polymerase σ32 factor	671099	+	869/894
	13	B4-1	ECH_0665	Phage uncharacterized protein	676091	−	907/1,410
	14	C2-1	ECH_0666	Adenosylmethionine-8-amino-7-oxononanoate aminotransferase	678007	−	1182/1,281
	15	A4-1	ECH_0669	3-Oxoacyl-(acyl-carrier-protein) reductase	683268	−	740/744
	16	s34 A2	ECH_0843	Recombination protein RecR	861495	+	581/588
	17	s33 E8	ECH_0878	Hypothetical protein	898815	−	456/1,230
	18	C4-2	ECH_1038	Hypothetical protein	1065224	−	5217/5,892
	19	A1-2	ECH_1067	d-Alanyl-d-alanine carboxypeptidase family protein	1095813	+	695/1,146
	20	s34 D8	ECH_1110	Dethiobiotin synthetase	1134587	−	98/693
	21	C3-2	ECH_1127	Major outer membrane protein OMP-1V	1150113	+	117/840
	22	B6-1	ECH_1144	Major outer membrane protein P28-1/OMP-20	1165318	−	712/816
*mCherry*	23	C2-3	ECH_0039	120-kDa immunodominant surface protein	34759	+	1305/1,647
	24	A4-3	ECH_0561	AcrB/AcrD/AcrF family protein	566002	+	1781/3,099
	25	D4-3	ECH_0837	tRNA-i(6)A37 modification enzyme MiaB	854867	−	56/1,329
	26	C6-3	ECH_0945	Hypothetical protein	964068	+	3741/4,050
	27	TR31	ECH_1144	Major outer membrane protein P28-1/OMP-20	1165587	+	444/816
*GFPuv*	28	D1-4	ECH_0104	Hypothetical protein	90734	+	89/126
	29	D5-4	ECH_0329	Hypothetical protein	317141	+	649/684
	30	D4-4	ECH_0666	Adenosylmethionine -8-amino-7-oxononanoate aminotransferase	676872	+	47/1,281
	31	D3-4	ECH_0866	Hypothetical protein	888668	+	651/993

a As per GenBank accession no. CP000236.

b The “+” and “−” refer to an insertion mutation in the same orientation of the ORF and in the opposite orientation, respectively.

**TABLE 2:** *E. chaffeensis* Himar1 transposon insertion mutants in intergenic region (total, 24)

Insertion cassette	Mutant no.	Mutant code	Gene no.^*a*^	Gene product name	Genomic insertion location	Flanking gene orientation^*b*^	Mutation distances to flanking up-/downstream ORFs (bp)
*mCherry loxP*	32	B6-2	ECH_0124/0125	Citrate synthase I/glutamate-cysteine ligase	113250	→ + →	294/10
	33	A3-1	ECH_0282/0283	Hypothetical protein/hypothetical protein	264190	→ + ←	492/737
	34	A2-1	ECH_0372/0373	Hypothetical protein/dihydroorotase	364550	→ + ←	356/159
	35	s34 D7	ECH_0537/0538	Arginyl-tRNA synthetase/isoleucyl-tRNA synthetase	539098	→ + ←	78/34
	36	s33 F3	ECH_0579/0580	Type IV secretion system protein VirB8/hypothetical protein	589238	→ − ←	46/424
	37	s33 B7	ECH_0593/0594	Hypothetical protein/acetylglutamate kinase	600196	← + ←	21/248
	38	D1-1	ECH_0605/0606	Glutamyl-tRNA synthetase/hypothetical protein	611987	→ + →	193/293
	39	s34 B1	ECH_0657/0658	tRNA-Ser/hypothetical protein	671821	← + ←	120/26
	40	s33 E7	ECH_0750/0751	DNA topoisomerase I/YjeF family protein	756968	→ − →	253/143
	41	D2-1	ECH_0769/0770	Exopolysaccharide synthesis protein/hypothetical protein	779062	← − ←	414/59
	42	B5-2	ECH_0930/0931	Putative BolA protein/pyridoxamine 5′-phosphate oxidase	953314	→ + ←	243/158
	43	s34 E1	ECH_1008/1009	Preprotein translocase, YajC subunit/DNA polymerase III, β subunit	1034973	→ + ←	119/10
	44	B6-2	ECH_1044/1045	Hypothetical protein/hypothetical protein	1076550	← + ←	1020/108
	45	A4-2	ECH_1065/1066	2-Oxoglutarate dehydrogenase/hexapeptide transferase family protein	1094096	← + ←	246/187
	46	s33 A7	ECH_1081/1082	SURF1 family protein/hypothetical protein	1109518	← + →	103/10
	47	D1-3	ECH_0083/0084	Hypothetical protein/hypothetical protein	74911	→ − →	83/386
	48	B2-3	ECH_0149/0150	Pyruvate dehydrogenase subunit beta/hypothetical protein	141187	← + ←	476/15
	49	B6-3	ECH_0579/0580	Type IV secretion system protein VirB8/hypothetical protein	589575	→ − →	383/87
*mCherry*	50	A3-3	ECH_0699/0670	Hypothetical protein/hypothetical protein	708076	→ + →	75/170
	51	A2-3	ECH_0705/0706	Peptide chain release factor 2/hypothetical protein	716170	→ − ←	63/49
	52	D5-3	ECH_0760/0761	RNA polymerase sigma factor RpoD/DNA primase	768120	→ + ←	61/387
	53	C6-3	ECH_0894/0895	Conserved domain protein/conserved hypothetical protein	920356	← + ←	133/133
	54	D6-3	ECH_0995/0996	Hypothetical protein/ATP-dependent protease peptidase subunit	1020175	→ − →	26/667
	55	B3-3	ECH_1148/1149	Hypothetical protein/preprotein translocase subunit SecA	1169030	→ − →	248/157

a As per GenBank accession no. CP000236.

b The “+” and “−” refer to insertion mutation in the forward orientation and reverse orientation, respectively.

### Impact of mutations on the RNA expression for gene disruption mutations and for the genes flanking insertion sites.

To assess the impact of the transposon insertion mutations on gene expression, reverse transcription-PCR (RT-PCR) analysis was performed targeting genes with open reading frame disruptions and the flanking genes for the intergenic sequence mutations. Transcriptional inactivation was observed for all gene disruption mutations downstream from the mutation insertion sites. For the intergenic sequence mutations, transcripts were detected for all genes located upstream and downstream of insertion sites when tested by RT-PCR in the subset of mutations assessed.

### *In vitro* growth defects assessed for the mutants.

**FIG 4:**
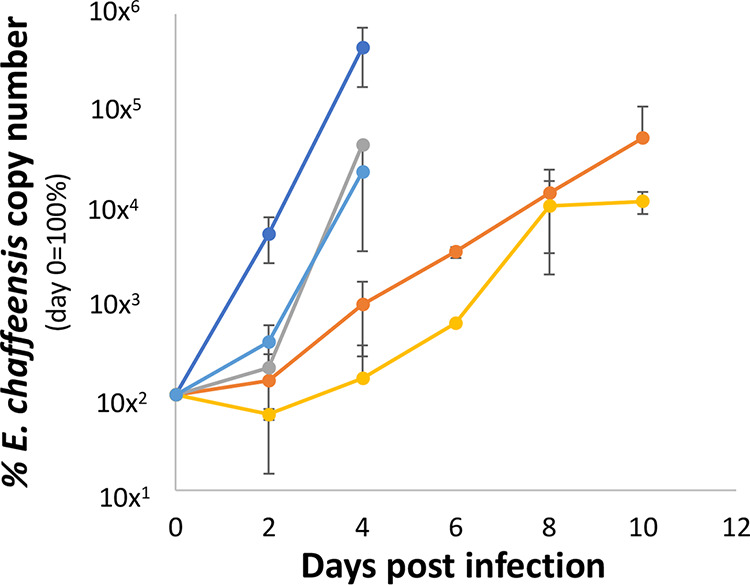
Culture assessment of four mutants having retarded growth in the canine macrophage cell line DH82. The growth of the mutants having insertion mutations in ECH_0039 (light blue line), ECH_0837 (yellow line), ECH_1127 (gray line), and ECH_1144 (orange line) is compared with that of wild-type *E. chaffeensis* (dark blue line). The number of bacteria at each time point postinoculation in DH82 was estimated by determining the copy numbers of the 16S rRNA gene. Each data point represents the average percentage of bacterial number relative to day 0 of inoculation from triplicate samples. The vertical bar represents standard deviation. *P* values were estimated by comparing the growth of the wild type with ECH_1127 (*P* = 0.06), ECH_0039 (*P* = 0.1), ECH_1144 (*P* = 0.04), and ECH_0837 (*P* = 0.04) on days 2 and 4.

As the mutagenesis experiments were executed in the ISE6 tick cell line, all identified mutants are considered to have no detrimental effect on *E. chaffeensis* growth in these cells. When we attempted to adapt the growth of the mutants to a macrophage-like cell line (DH82), four mutants displayed delayed growth compared to the wild-type *E. chaffeensis*, while the remaining mutants grew similarly to the wild type. The mutants with disruption of gene activities for ECH_0837, encoding the tRNA-i(6)A37 modification enzyme MiaB (a metal ion binding protein), and ECH_1144, encoding a member of the 28-kDa outer membrane protein (P28/OMP) gene family, P28-1/OMP-20, had significantly slower growth in the macrophage cell line (DH82) as assessed by comparing the growth of wild-type *E. chaffeensis* (Fig. 4). The mutants with insertions into ECH_1127 (the gene encoding another P28/OMP protein [OMP-1V/OMP-6]) and ECH_0039 (the gene encoding the 120-kDa immunodominant surface protein) had an initial lag phase in DH82 cell culture but recovered thereafter.

### Infection study in the canine host to define the importance of *E. chaffeensis* genes critical for the pathogen’s persistent growth.

**FIG 5:**
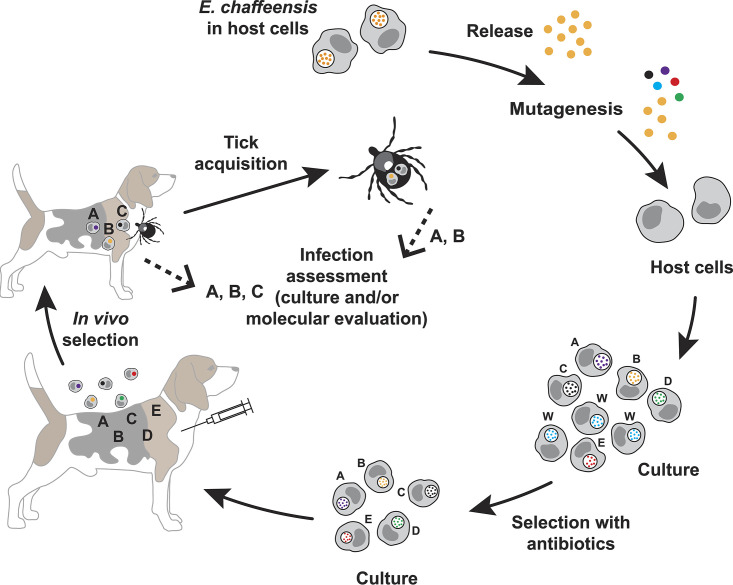
Schematic representation of *E. chaffeensis* transposon mutants assessed to identify genes important for the pathogen’s *in vivo* growth. The method involves recovering ISE6 culture-derived *E. chaffeensis* organisms, subjecting them to transposon mutagenesis to identify mutants in cultures resistant to antibiotic clearance, infecting the canine host, and acquisition feeding assessment of *A. americanum* ticks.

We investigated how mutations within both coding and noncoding regions impacted *E. chaffeensis* growth and persistence in a mammalian host and their acquisition from the host by *A. americanum*. A physiologically relevant canine host model was infected with pools of mutants and assessed for the presence and absence of mutants circulating in the blood of infected animals (Fig. 5). Xenodiagnosis was also performed using *A. americanum* ticks. This approach was similar to that described in our prior studies, which aided in mapping genes required for *E. chaffeensis* persistent growth (14). Similar methods have been employed in defining virulence-associated proteins for several other pathogenic bacteria (29–31). In the current study, we followed the same strategy as in our prior investigations of infection assessment of *E. chaffeensis* mutants (14). We used three beagle dogs per pool of randomly selected mutants; about equal numbers of mutants with gene disruption mutations and those with insertions into intergenic spaces were used in each pool. A total of 51 mutants were tested in 6 infection pools; 2 pools each containing 8 and 9 mutants and 1 pool each with 7 and 10, respectively. (Mutant numbers 2, 9, 36, and 44 [listed in Tables 1 and 2] were not part of the infection experiment.) *In vitro* cultures of mutants were mixed in each pool with approximately equal numbers of mutant organisms and used as infection inocula. Blood was sampled twice a week for 8 weeks from all dogs and used to assess infection by *aadA*-specific PCR and by performing insertion region-specific PCR analysis to detect mutants. Similarly, several tissue samples were assessed at the terminal point of the study (after 8 weeks). To assess if the mutants persisting in the canine host were acquired by a tick host (*A. americanum*), flat nymphal ticks were allowed to acquisition feed to repletion on dogs starting from day 5 postinfection, which lasted until about day 12. Following molting, adult ticks of both sexes were randomly selected and evaluated individually for the presence of mutants by performing insertion-specific and *aadA* gene-specific PCR assays to detect the mutants acquired by *A. americanum* (Table 3).

**TABLE 3:** Mutants that persisted in the canine host

Mutant no.	Mutant code	Gene no.	Protein identifier	Dogs	No. of ticks positive/no. tested
12	B1-2^*a*^	ECH_0655	RNA polymerase σ32 factor	+ − −	0/10
13	B4-1	ECH_0665	Phage uncharacterized protein	+ + +	2/10
17	S33 E8	ECH_0878	Hypothetical protein	+ + −	7/17
18	C4-1^*a*^	ECH_1038	Hypothetical protein	+ − −	0/10
19	A1-2	ECH_1067	d-Alanyl-d-alanine carboxypeptidase family protein	+ + +	3/10
26	C6-3	ECH_0945	Hypothetical protein	+ + +	0/17
34	A2-1	ECH_0372/0373	Hypothetical protein/dihydroorotase	+ + +	4/17
38	D1-1^*a*^	ECH_0605/0606	Glutamyl-tRNA synthetase/hypothetical protein	+ + +	0/10
40	s33 E7	ECH_0750/0751	DNA topoisomerase I/YjeF family protein	+ − −	2/17
45	A4-2	ECH_1065/1066	2-Oxoglutarate dehydrogenase/hexapeptide transferase family protein	+ + +	5/10
48	B2-3^*a*^	ECH_0149/0150	Pyruvate dehydrogenase subunit beta/hypothetical protein	+ + −	0/10
53	C6-3^*a*^	ECH_0894/0895	Conserved domain protein/conserved hypothetical protein	+ + +	0/10
55	B3-3	ECH_1148/1149	Hypothetical protein/preprotein translocase subunit SecA	+ + +	1/10

a Tested negative by xenodiagnosis.

All dogs tested positive for the *aadA* gene several times throughout the study period in blood and also in several tissue samples at the study’s terminal time point, suggesting the persistence of one or more mutant organisms used in each pool. PCR analysis targeting specific mutants using the respective mutant insertion-specific PCRs resulted in the identification of only a subset of mutants in each pool. If a mutant was detected in blood after a week for at least one of the three dogs and/or in one or more tissue samples, then the respective insertion mutation was considered to have minimal impact on the persistence of *E. chaffeensis* growth (Table 3). If a mutant was not detected in dogs any time during the 8-week study period and was also negative in tissue samples, then the mutation was regarded as detrimental for the pathogen’s persistence in the canine host. Thirteen mutants persisted in the canine host, which included 6 gene disruption mutations and 7 mutations within the intergenic spaces (Table 3). The persisting mutants included three each with gene disruptions in hypothetical protein genes and in genes with predicted annotated protein names. The only notable gene disruption mutant organism that persisted was with a mutation in the gene for RNA polymerase sigma factor σ32 (RpoH) (ECH_0655). Insertion in this gene was located near the 3′ end of the open reading frame, 26 nucleotides upstream from the stop codon (Table 1). Eight of the 13 persistent mutants were also detected in ticks allowed to feed on the dogs (Table 3). Of the five persistent mutants which tested negative by xenodiagnosis, two were gene disruptions; one each in a hypothetical protein gene (ECH_1038) and in the RpoH gene and the remaining three are located within the intergenic spacers (mutant numbers 38, 48, and 53) (Table 3).

There were significantly more rapidly cleared mutant organisms than those that persisted (Table 3). The rapidly cleared mutants included 23 with gene disruption mutations (Table 4) and 15 with insertion mutations in noncoding regions (Table 5). The gene disruption mutations included mutations in genes encoding 9 hypothetical proteins, an oxidoreductase family protein, two proteins each involved in the protein synthesis machinery and biosynthesis of biotin, one protein each with a role in fatty acid metabolism, a multidrug resistance (MDR) efflux pump protein, a DNA repair protein, three immunodominant outer membrane proteins (two belonging to the P28/OMP protein family and one representing the 120-kDa immunodominant surface protein), and a metal ion binding protein (tRNA-i[6]A37 modification enzyme protein [MiaB]). Several intergenic spacer mutations were also identified with insertion mutations near genes likely engaged as virulence determinants. They included genes encoding various synthases and proteins involved in transcription and translation. Three mutants tested positive by xenodiagnosis, while they were undetected in the canine host. They included one gene disruption mutation in a hypothetical protein gene (ECH_0113) and two within the noncoding regions (mutants 46 and 47) (Table 4). It is likely that these mutants may have circulated in the canine host at a very low level.

**TABLE 4:** *E. chaffeensis* gene disruption mutants cleared from the canine host

Mutant no.	Mutant code	Gene no.	Protein identifier
1	A1-1^*a*^	ECH_0113	Hypothetical protein
3	D4-1	ECH_0242	Hypothetical protein
4	s33 E5	ECH_0251	Hypothetical protein
5	D3-2	ECH_0368	Dioxygenase family protein (oxidoreductase)
6	s34 C8	ECH_0445	Queuine tRNA-ribosyltransferase (protein synthesis)
7	C1-1	ECH_0475	Signal recognition particle protein (protein synthesis)
8	s33 C7	ECH_0525	Hypothetical protein
10	B5-1	ECH_0600	Hypothetical protein
11	D3-1	ECH_0614	Hypothetical protein
14	C2-1	ECH_0666	Adenosylmethionine-8-amino-7-oxononanoate aminotransferase (biotin biosynthesis)
15	A4-1	ECH_0669	3-Oxoacyl-(acyl-carrier-protein) reductase (fatty acid biosynthesis)
16	s34 A2	ECH_0843	Recombination protein RecR (DNA repair)
20	s34 D8	ECH_1110	Dethiobiotin synthetase (biotin biosynthesis)
21	C3-2	ECH_1127	Major outer membrane protein OMP-1V/OMP-6 (immunogenic outer membrane protein)
22	B6-1	ECH_1144	Major outer membrane protein P28-1/OMP-20 (immunogenic outer membrane protein)
23	C2-3	ECH_0039	120-kDa immunodominant surface protein (immunogenic outer membrane protein)
24	A4-3	ECH_0561	AcrB/AcrD/AcrF family protein (MDR efflux protein)
25	D4-3	ECH_0837	tRNA-i(6)A37 modification enzyme MiaB (metal ion binding/tRNA modification)
27	TR31	ECH_1144	Major outer membrane protein P28-1/OMP-20 (immunogenic outer membrane protein)
28	D1-4	ECH_0104	Hypothetical protein
29	D5-4	ECH_0329	Hypothetical protein
30	D4-4^*b*^	ECH_0666	Adenosylmethionine-8-amino-7-oxononanoate aminotransferase (biotin biosynthesis)
31	D3-4	ECH_0866	Hypothetical protein

a Tested positive by xenodiagnosis.

b D4-4 tested positive only in tissue samples of two animals on day 56.

**TABLE 5:** *E. chaffeensis* intergenic mutants cleared from the canine host

Mutant no.	Mutant code	Gene no.	Protein identifier
32	B6-2	ECH_0124/0125	Citrate synthase I/glutamate-cysteine ligase
33	A3-1	ECH_0282/0283	Hypothetical protein/hypothetical protein
35	s34 D7	ECH_0537/0538	Arginyl-tRNA synthetase/isoleucyl-tRNA synthetase
37	s33 B7	ECH_0593/0594	Hypothetical protein/acetylglutamate kinase
39	s34 B1	ECH_0657/0658	tRNA-Ser/hypothetical protein
41	D2-1	ECH_0769/0770	Exopolysaccharide synthesis protein/hypothetical protein
42	B5-2	ECH_0930/0931	Putative BolA protein/pyridoxamine 5′-phosphate oxidase
43	s34 E1	ECH_1008/1009	Preprotein translocase, YajC subunit/DNA polymerase III, β subunit
46	s33 A7^*a*^	ECH_1081/1082	SURF1 family protein/hypothetical protein
47	D1-3	ECH_0083/0084	Hypothetical protein/hypothetical protein
49	B6-3^*a*^	ECH_0579/0580	Type IV secretion system protein VirB8/hypothetical protein
50	A3-3	ECH_0699/0670	Hypothetical protein/hypothetical protein
51	A2-3	ECH_0705/0706	Peptide chain release factor 2/hypothetical protein
52	D5-3	ECH_0760/0761	RNA polymerase sigma factor RpoD/DNA primase
54	D6-3	ECH_0995/0996	Hypothetical protein/ATP-dependent protease peptidase subunit

a Tested positive by xenodiagnosis.

## DISCUSSION

*E. chaffeensis* is among several tick-transmitted rickettsial bacteria responsible for causing zoonosis in people. Despite several recent advances in performing molecular genetic studies, only limited progress is documented in understanding pathogenesis and in identifying rickettsial proteins posited to contribute to bacterial virulence and host-pathogen interactions and those involved in supporting the immune evasion mechanisms (14, 16, 20, 23, 24, 32–34). One of the challenges is that the pathogenic organisms are difficult to grow in axenic culture media (35). Furthermore, the mutational success rate remains extremely limited even with Himar1 mutagenesis plasmids, possibly because *Ehrlichia* and *Anaplasma* species are not known to naturally harbor extrachromosomal plasmids. For example, several attempts are required to generate a moderately sized mutant library for *Ehrlichia* and *Anaplasma* species, and often none to only a few mutant organisms are recovered following a typical mutational experiment (14, 19, 23, 24). We previously reported 9 random mutations within the *E. chaffeensis* genome in our prior study (14). Subsequent research has focused on detailed characterization of three mutant *E. chaffeensis* organisms (16, 17, 36, 37). These studies aided in the identification of one mutation in the ECH_0660 gene causing the rapid clearance of the pathogen and inducing a sufficient host immune response to serve as a live attenuated vaccine to protect against wild-type infection challenge by intravenous (i.v.) injection, as well as by tick transmission (16, 17). Mutagenized *E. chaffeensis* organisms are also valuable in studies focused on understanding the host response against the pathogen (17, 18).

In this study, we carried out investigations in generating a mutant library consisting of 55 mapped insertion mutations within the *E. chaffeensis* genome. We used three different Himar1 mutagenesis constructs for generating the mutational library. Further, several independent mutagenesis experiments were performed to generate more mutations in the *E. chaffeensis* genome. While all constructs worked similarly in producing a low mutation rate, there was no bias observed toward generating gene open reading frame disruption mutations or intergenic mutations, as nearly equal numbers of intergenic and intragenic mutations were observed. Our previously well-defined physiologically relevant canine infection model, molecular assessment, and xenodiagnosis methods (14–17, 38, 39) were valuable in the current study to identify many *E. chaffeensis* gene sequences associated with persistent infection of dogs. Of the 29 gene open reading frame disruption mutations tested, only 6 mutants (21%) persisted in the host, while mutations in the remaining 23 mutants (79%) were rapidly cleared. These data suggest that disruptions in the majority of the pathogen genes can be detrimental to the pathogen’s persistence *in vivo* and that the disrupted genes causing the rapid clearance are among the many essential genes of *E. chaffeensis*. Nine of the 23 genes identified as critical for *E. chaffeensis* persistent growth encode hypothetical proteins. The list of essential genes included two coding for proteins known to be involved in protein synthesis machinery, three coding for outer membrane-expressed immunogenic proteins, two genes belonging to biotin biosynthesis, and one each representing the DNA repair machinery, fatty acid biosynthesis, MDR efflux pump, and an oxidoreductase (dioxygenase family protein; ECH_0368). Rapid clearance of *E. chaffeensis* from the canine host resulting from the gene function disruption mutations suggests that the pathogen has retained many important pathway genes to support its obligate parasitic lifestyle.

Outer membranes of Gram-negative bacteria contain many proteins that perform essential functions, such as for the nutrient uptake mediated by porin activities, cell adhesion, cell signaling, and waste export, as well as to support the evasion of host defense mechanisms by pathogenic bacteria (40). Several P28 outer membrane proteins (P28/OMP) encoded from a multigene locus are identified as immunodominant proteins in *E. chaffeensis* (41–47). Similarly, the 120-kDa surface protein (also known as TRP 120) is recognized as an immunodominant protein of *E. chaffeensis* (41–47). Mutations reported in the current study included two P28/OMP genes (p28-1/OMP-20 and OMP-1v/OMP-6) and TRP 120. Disruption mutations in these three immunogenic outer membrane protein genes also caused *in vitro* growth defects in DH82 cultures, suggesting that the membrane proteins are essential for the pathogen’s replication in macrophages both *in vitro* and *in vivo*. The discovery of the two P28/OMP proteins and TRP 120 as essential for *E. chaffeensis* persistent growth is consistent with the prior studies demonstrating the importance of these immunogenic outer membrane-associated proteins for the pathogen’s replication in macrophages *in vitro* and *in vivo*. Indeed, two *E. chaffeensis* P28/OMPs (P28/OMP-19 and OMP-1F/OMP-18) have been reported to possess porin-like structures and porin activities (48). Further to this, two independent studies demonstrated that antibodies targeting P28/OMP-19 can block the infection progression *in vivo* and *in vitro* (41, 49). TRP 120 has been extensively investigated by the McBride group for its multiple roles, with recent evidence pointing to the protein being likely essential for the continued replication of *E. chaffeensis* in phagosomes (47). *E. chaffeensis* TRP 120 is expressed on the cell surface as well as a type 1 secretory system-mediated translocated effector having several defined functions, such as being engaged in the pathogen’s host cell entry (46), as a host cell nuclear translocation to serve as a nucleomodulin in regulating gene expression associated with signal transduction and apoptosis (50), and in interacting within the host cytoplasm as a moonlighting effector, a ubiquitin ligase targeting host nuclear proteins (47).

We expected that that the disruption mutation in the RpoH gene encoding the RNA polymerase sigma factor (σ32) would be detrimental for *E. chaffeensis*, but this mutant persisted, although it was detected less frequently and tested negative by xenodiagnosis. Two possibilities for this outcome are that (i) the *E. chaffeensis* σ70 protein may have complemented the function of σ32 in the mutant and (ii) a modified but functional version of the protein lacking the last 8 amino acids is formed. The first hypothesis is supported by our previous study demonstrating that *E. chaffeensis* gene promoters can be recognized by RNA polymerase holoenzyme containing either σ32 or σ70 in initiating transcription from a gene (51). The mutant tested positive for the RpoH transcript when assessed by RT-PCR targeting the region upstream of the mutation insertion site (data not shown). Thus, assuming that the truncated version of the transcript is translated, it is also highly likely that the mutated version of the protein is functionally active.

The current study is the first to demonstrate that mutations in two different biotin pathway enzyme genes have similar impacts on rapidly clearing a rickettsial pathogen from the vertebrate host. The *E. chaffeensis* genome includes the biotin biosynthesis pathway protein genes (52–54). Similarly, biotin pathway genes are conserved in other related rickettsiae (53, 55). A recent study suggested that *E. chaffeensis* biotin pathway genes are functionally active, as judged from experiments performed using the Escherichia coli complementation system (52). Biotin is an essential cofactor for several key metabolic pathways in bacteria, such as fatty acid biosynthesis and amino acid metabolism (56, 57). Indeed, many microorganisms synthesize biotin *de novo* (56, 58–60). Considering the absence of biotin synthesis machinery in mammals (56, 61), *E. chaffeensis* and other related *Rickettsiales* may have evolved to maintain their own functional biotin synthesis pathway. The biotin pathway enzymes are also attractive targets in generating antibacterial inhibitors (62, 63). Considering the availability of only one class of drugs (tetracycline derivatives) to treat rickettsial infections (64, 65) and that doxycycline-treated patients may remain persistently infected with *Ehrlichia* species (66, 67), studies may be extended in developing novel drugs targeting the biotin synthesis pathway.

We also discovered that the mutation in a fatty acid biosynthesis gene, 3-oxoacyl-(acyl-carrier-protein) reductase gene, results in *E. chaffeensis* rapid clearance from the vertebrate host. Similarly, MDR efflux pump, DNA repair, and protein synthesis pathway proteins are among the proteins essential for *E. chaffeensis* persistent growth *in vivo*. The efflux pump is known to play a critical role in conferring resistance to antibiotics in several bacteria (68–70). Efflux pump proteins are, therefore, commonly known as a requirement for bacterial virulence and also serve as an attractive target for designing novel therapeutics (68–70). It is not yet defined if *E. chaffeensis* has an active efflux pump to eliminate antibiotic accumulation from its cytoplasm. Reflecting on the essential nature of the identified efflux pump protein of the pathogen, it is highly likely that the human monocytic ehrlichiosis agent uses this protein for the benefit of clearing host defense proteins from its cytoplasmic space.

As with gene disruption mutants, only about one-third of intergenic spacer mutations (7 of 22) were identified as nonessential, while the majority (15 of 22) were among the rapidly cleared mutants. This is a surprising outcome, as the mutations did not appear to impact the transcription of the genes upstream and downstream from the insertion sites. However, in our prior studies, we reported that intergenic mutations can cause polar effects in altering the gene expression from genes located proximal to the mutation insertion sites (38). Interestingly, mutations in genomic regions found to be essential for the pathogen’s *in vivo* growth included several genes upstream and downstream of the insertion sites coding for proteins likely involved in the protein synthesis machinery and DNA replication and transcription and type IV secretion system-associated proteins. While it is unclear how the intergenic mutations impact the expression of genes proximal to insertion mutations, it is conceivable that the mutations can alter gene expression and potentially render the transcripts less stable, thus interfering with protein synthesis. Also, we cannot rule out the possibility that mutations within intergenic spacers may have impacted gene expression by disrupting the normal function of regulatory elements, including those involving the contributions of microRNAs. Several recent studies described the existence of microRNAs in *Rickettsiales* having a functional role in bacterial gene regulation (71, 72).

Five mutant organisms persisted in the canine host, while they were undetectable by xenodiagnoses in ticks. It is likely that the mutations caused defective growth of *E. chaffeensis* in its tick vector. Similarly, several genes/genomic regions found to be critical for the pathogen persistence in the canine host may also be critical for its replication in the tick host. This hypothesis remains to be tested. We recently described a needle inoculation method of infecting ticks, which bypasses the need for tick infection acquisition from a vertebrate host (39). This method will be valuable in determining which genomic regions of the pathogen are critical for *A. americanum* to harbor *E. chaffeensis* infection.

The current study demonstrates that Himar1 mutagenesis and *in vivo* screening methods using a physiologically relevant incidental host are ideally suited for mapping many essential bacterial proteins associated with *E. chaffeensis* virulence and persistent growth. The discovery of many genes essential for the continuous *in vivo* growth of *E. chaffeensis* opens the path for studies to define pathogenesis and develop novel therapeutics targeting critical pathways of the organism and also to extend such studies to other important *Ehrlichia* and *Anaplasma* pathogens impacting human and animal health.

## MATERIALS AND METHODS

### *E. chaffeensis in vitro* cultivation.

Wild-type *E. chaffeensis* isolate Arkansas and the mutated organisms were continuously cultivated in an Ixodes scapularis cell line (ISE6) at 34°C in the absence of CO_2_ (73). Where applicable, the organisms were also cultivated in a canine macrophage cell line (DH82) at 37°C with 5% CO_2_ as described earlier (74, 75).

### Generation of *E. chaffeensis* transposon mutant library.

Three different plasmid constructs encoding the Himar1 transposase, antibiotic resistance conferred by *aadA*, and a fluorescent protein (mCherry or GFP) driven by the Anaplasma marginale Am-tr promoter were used for mutagenesis of *E. chaffeensis*: (i) pCis mCherry-SS Himar A7 containing the *mCherry* and *aadA* genes (14, 25), (ii) pHimar1 A7 loxP plasmid containing the *mCherry* and *aadA* genes flanked by *loxP* sites, and (iii) pCis GFPuv-SS Himar A7 containing the *gfpuv* and *aadA* genes (14, 27). The pHimar1 A7 loxP plasmid was generated by inserting mismatched *loxP* sites (76, 77) flanking the transposon segment into pCis mCherry-SS Himar A7. Host cell-free *E. chaffeensis* suspensions recovered from ISE6 tick cell cultures were subjected to mutagenesis by following the protocol we described earlier (14, 15). Briefly, a 5-ml culture of *E. chaffeensis*-infected ISE6 cells was transferred to microcentrifuge tubes containing 0.2 ml of silicon carbide (no. 1 coarse rock tumbling grit; Loretone Inc., Mukilteo, WA) and vortexed for 30 s at high speed. The supernatant was passed through a 2-μm-pore-size filter (Whatman Ltd., Piscataway, NJ), and bacteria were collected by centrifugation at 11,000 × *g* at 4°C for 5 min. Bacteria were washed twice in 0.3 M sucrose and kept on ice between washes. Aliquots of purified *E. chaffeensis* (∼5 × 10^8^) were resuspended in 50 μl of cold 0.3 M sucrose containing 1 μg of plasmid DNA, transferred to a 1-mm-gap electroporation cuvette, and incubated on ice for 15 min (19). (Plasmid DNAs were prepared using a Maxiprep plasmid DNA isolation kit by following the manufacturer’s instructions [Qiagen, Valencia, CA.]) *E. chaffeensis* organisms were electroporated at 2,000 V, 25 μF, and 400 Ω. The mixture was then combined with 0.5 ml of fetal bovine serum and 1 ml of ISE6 cell suspension containing about 1 × 10^6^ cells. The sample was centrifuged at 5,000 × *g* for 5 min, incubated at room temperature for 15 min, and then mixed with ISE6 cells from a confluent culture (∼1 × 10^7^ cells). Cells were then seeded into all wells of a 48-well plate, incubated at 30°C overnight, and then transferred to a 34°C incubator for ISE6 cells for the continuous growth of the organisms. After 48 h, 100 μg/ml each of spectinomycin and streptomycin was added to the culture medium to select mutants. The culture medium containing antibiotics was replaced once a week. When infectivity reached 80% or higher, cell-free *Ehrlichia* was prepared for inoculating a new flask of uninfected host cells with medium containing antibiotics. This procedure was repeated until all wild-type bacteria were eliminated. The presence of insertion mutations was monitored for 60 days or longer.

### Southern blot analysis to identify mutations in *E. chaffeensis*.

Genomic DNA from the transformant cultures was isolated using a genomic DNA isolation kit as per manufacturer instructions (Qiagen, Valencia, CA). About 100 ng of genomic DNA recovered from cultured organisms was digested with BglII restriction enzyme for 2 to 3 h at 37°C. The digested DNA samples were resolved on a 0.9% agarose gel for about 6 h at 60 V and transferred to a nylon membrane. Blots were then hybridized with a ^32^P-labeled *aadA* gene probe at 68°C overnight, followed by washing steps to identify specific DNA-probe interactions as per our previously described protocol (14). The hybridized membranes were exposed to X-ray film to observe radioactive signals emitting from hybridized blots.

### Mapping and verification of transposon insertions.

The genomic locations of the insertions within the mutated bacteria were mapped with the help of a Universal Genome Walker 2.0 kit (Clontech Laboratories). SspI restriction enzyme was used for DNA fragmentation and for genomic library construction. Inserted fragment-specific primers for PCR amplifications were GSP1 and GSP2. A third primer, GSP3, was used to sequence the PCR products (primers are listed in Table S1). Sequence data were then subjected to BLAST search analysis to localize the insertion sites within the *E. chaffeensis* genome (GenBank accession no. CP000236.1). Subsequently, inserted fragment-specific PCRs were performed using primers targeting genomic regions either 5′ or 3′ to insertion sites and to an inserted fragment-specific sequence. The primers targeting the genomic region of each mutant are listed in Table S1; the inserted fragment-specific primers are Amtr R1 (RG92), mCherry R1 (RG97), and aadA F1 (RG1202). The expected PCR product sizes are indicated in Table S1.

### Transcriptional analysis to assess the impact of mutations.

Total RNAs from *E. chaffeensis* mutants grown in ISE6 cell cultures were isolated using the Tri-reagent RNA isolation method as per the manufacturer’s instructions (Sigma-Aldrich, St. Louis, MO). Total RNA was treated with RQ1 DNase at 37°C for 60 min to remove any genomic DNA contamination. Primers targeting each insertion-specific mutation were designed for use in RT-PCR analysis. For each set of RT-PCRs, controls included reactions without reverse transcriptase, reactions with genomic DNA as a template, or reactions with no DNA or RNA added. For verifying RNA expression, the presence or absence of specific products in the assays containing RNA with reverse transcriptase was assessed and compared with the products generated from genomic DNA-positive controls. Similarly, RT-PCR assays were performed to assess transcriptional changes from genes upstream and downstream from insertion sites for the insertion mutations located in the intergenic spaces. (All RT-PCR primers are listed in Table S1.)

### *In vitro* growth analysis of mutants in the canine macrophage cell line DH82.

To assess the effect of different gene mutations on *E. chaffeensis* growth in macrophage cells, we attempted to regrow mutants in the canine macrophage cell line DH82, as described earlier (14). Nearly all mutants could be cultured in DH82 cells and exhibited growth patterns similar to that of wild-type *E. chaffeensis*, except for a few mutant organisms. The growth of mutants having insertions in certain genes was considerably retarded in DH82 cells. To further assess the growth variations of the slow-growing mutants compared to the wild type, cell-free bacteria recovered from about 90% infected 25-cm^2^ flasks from slow-growing mutants and wild-type *E. chaffeensis* were recovered from ISE6 cell cultures and used to assess their growth in DH82 cells. Briefly, bacteria from infected ISE6 cells were recovered by repeated passing the cultures through a bent 27-gauge needle and then filtered using a 2-μm filter. Cell-free bacteria from the filtrate were then recovered following centrifugation at 10,000 × *g* for 5 min. The pellets were resuspended in 3 ml each of culture medium. About 100 μl of the cell-free bacteria was then used to infect confluent DH82 monolayers in 12-well plates in triplicates for each mutant. After 24 h, the monolayers were washed to remove any cell-free bacteria. The cultures were monitored for up to 12 days. At 2-day intervals, the cultures were recovered from the individual triplicate wells representing each mutant or the wild type, and DNAs were purified and then assessed for bacterial growth by quantitative PCR. Bacterial numbers were estimated by real-time quantitative PCR targeting the16S rRNA gene segment, as we described previously (78). This experiment was performed three independent times and using data collected from triplicate well samples each time.

### Dog infections with pools of *E. chaffeensis* mutants.

Animal experiments with dogs were performed in compliance with the Public Health Service (PHS) Policy on the Humane Care and Use of Laboratory Animals (https://olaw.nih.gov/policies-laws/phs-policy.htm), the U.S. Department of Agriculture’s (USDA) Animal Welfare Act & Regulations, and with the prior approval of the university Institutional Animal Care and Use Committee (IACUC). At the end of each experiment, all animals were euthanized in accordance with the IACUC recommendations, which are consistent with the recommendations of the Panel on Euthanasia of the American Veterinary Medical Association.

About 6-month-old female beagle dogs were obtained from a USDA-certified commercial breeder. Dogs were housed indoors at a climate-controlled animal facility at Kansas State University and *ad libitum* feed and water were provided. All dogs were placed in individual housing pens with adequate space to allow regular exercise/activity. In addition, all dogs were permitted to socialize in groups several times each day. The animals were also monitored daily for health and behavioral changes and twice weekly for body temperature and hematological changes during the study period. Veterinary care for the animals was overseen by a university veterinarian.

*E. chaffeensis* mutants grown in ISE6 cultures to about 80 to 90% infection in T75 flasks were harvested by centrifugation at 15,000 × *g* for 10 min at 4°C, supernatants were discarded, and the cultures were resuspended in 15 ml of 1× phosphate-buffered saline (PBS). The washing steps were repeated twice, and the final cell pellet was suspended to concentrate the infected ISE6 cells to about 2 × 10^6^ per ml, yielding an estimated concentration of *Ehrlichia* organisms of ∼2 × 10^8^ per ml. Equal volumes of the culture suspensions of randomly selected mutants were mixed for preparing mutant pools having equal ratios of the mutants in each pool. One milliliter of each mutant pool per dog was inoculated by i.v. injection.

### Evaluation of canine blood samples over time for the presence of mutants.

About 2 ml of blood was recovered from all dogs into sterile EDTA tubes on day 0 (prior to infection) and twice a week starting from the day 3 postinfection and until the end of 8 weeks. The blood samples were used immediately or stored at 4°C until use (maximum of 1 day). The samples were centrifuged at 3,000 rpm in a Clay Adams Sero-fuge (Becton, Dickinson, Sparks, MD) for 5 min, and buffy coats were transferred to a 15-ml sterile Falcon centrifuge tube containing 10 ml of erythrocyte (RBC) lysis buffer (155 mM NH_4_Cl, 10 mM KHCO_3_ and 0.1 mM EDTA) and mixed several times until complete lysis of erythrocytes. The samples were then centrifuged at 5,000 × *g* for 5 min. The buffy coat pellet from each sample was mixed in 300 μl of 1× PBS. One-hundred-microliter volumes of the buffy coats recovered from blood samples were used to recover total genomic DNA using the DNeasy blood and tissue kit (Qiagen, Germantown, MD). Purified DNA from each sample was dissolved in 200 μl of elution buffer. The DNAs were used to assess *E. chaffeensis* infection status by performing nested PCR targeting the inserted fragment-specific spectinomycin resistance gene (*aadA*) (primers for this experiment are listed in Table S1) as we described previously (14). Samples testing positive for the *aadA* gene were subsequently evaluated by nested PCRs targeting the transposon insertion fragment and the respective flanking genomic regions for the mutants using the insertion-specific primer sets (primers listed in Table S1).

### Xenodiagnosis of *E. chaffeensis* mutants by *A. americanum*.

About 200 each of the laboratory-reared nymphal *A. americanum* ticks (Ecto Services, Inc., Henderson, NC) were placed per each dog starting day 5 postinoculation. The ticks were allowed to complete the blood acquisition (about 7 days) and the recovered fed ticks were kept at room temperature and 14 h of light in a 96% humidity chamber for molting to the adult stage (which took between 36 and 50 days). Genomic DNAs from about 10 to 20 ticks (from each group of dogs) were isolated individually using the DNeasy blood and tissue kit (Qiagen). Purified DNA from each tick was resuspended in 200 μl of elution buffer. Two microliters of DNA derived from each tick was used for nested PCR analysis targeting the *aadA* gene, and those testing positive for the *aadA* gene were then retested for transposon insertion regions specific for each mutant, as described above.

### Statistics.

Statistical analysis was carried out to assess differences in average copy numbers of bacteria present in the wild type and mutants at each time point following *in vitro* growth in DH82 cells. The analysis was performed using the 2-tailed unpaired Student *t* test (GraphPad Software, La Jolla, CA).

## Supplementary Material

Supplemental file 1
