# Supplementary material for: Multiple Ehrlichia chaffeensis Genes Critical for Its Persistent Infection in a Vertebrate Host Are Identified by Random Mutagenesis Coupled with In Vivo Infection Assessment
Source: Infect Immun. 2020 Sep 18;88(10):e00316-20. doi: 10.1128/IAI.00316-20 (PMC7504954; doi:10.1128/IAI.00316-20)
Supplement: Supplemental file 1 [file IAI.00316-20-s0001.pdf]

Figure S1: Mapped mutants identified on the Southern blots

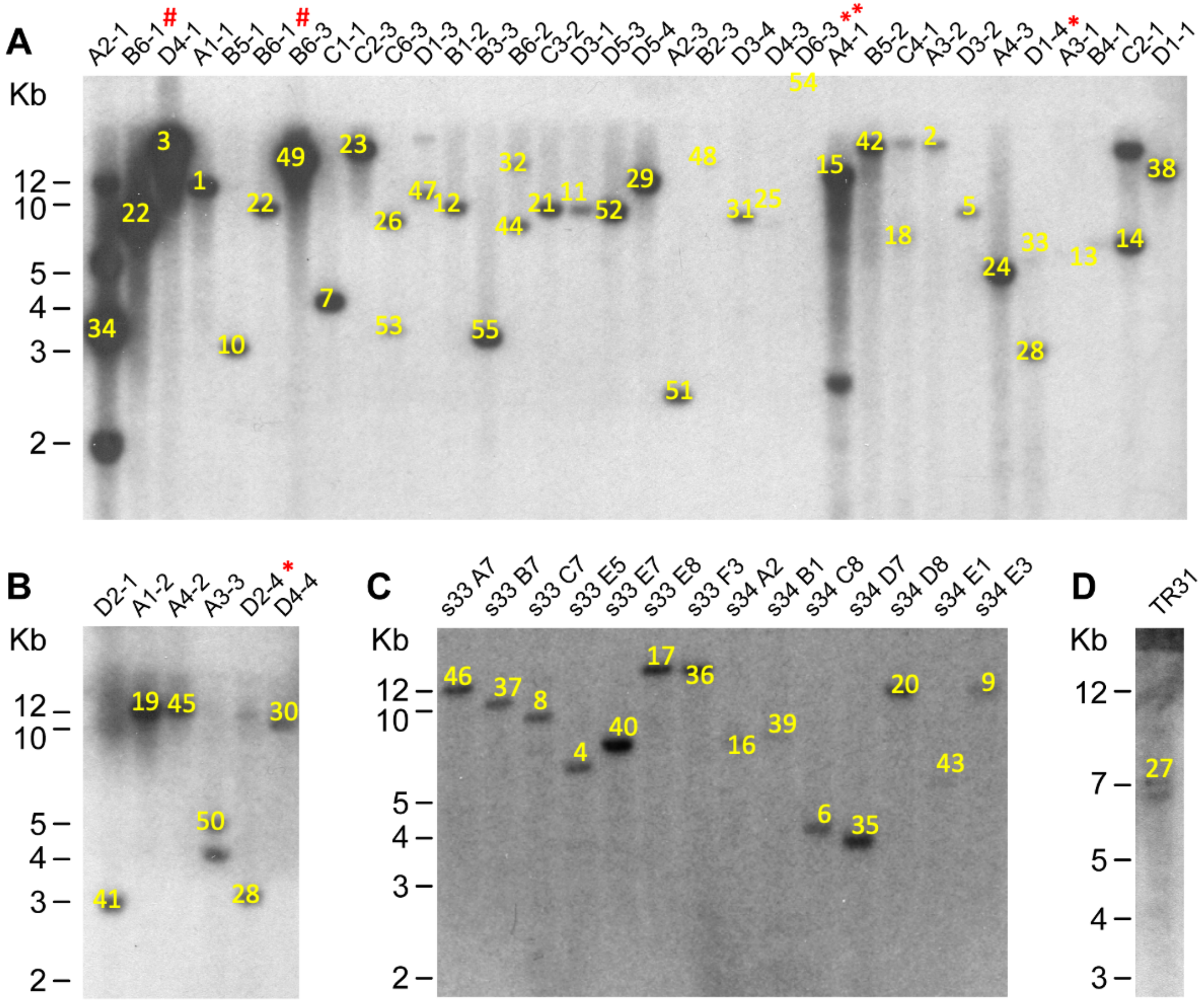

*D2-4 and D1-4 are identical; possible was the result of clonal duplication

**D6-3 tested negative by Southern blot, however, tested positive by insertion-specific PCR analysis.

#B6-1 was used twice in the Southern blot

Table S1: List of oligonucleotides used in this study

Mutant code	Locus	Name	Sequence (5' to 3')	Size(bp)
Primers used for genome mapping of the mutants:				
		GSP1	TGCTGGTTACGGTGACCGTAAGGCTTG	
		GSP2	TGCAGGTATCTTCGAGCCAGCCACGATC	
		GSP3	CAGTTGGAAGAATTTGTTCACTACGTG	
PCR primers for mutation verifications:				
A1-1	ECH_0113 ORF	RG254	CCGTTATCACATCAACTCCT	380
	(Amtr R1)	RG92	TGTTAGTGCGCGCGTTTTAT	
A1-2	ECH_1067 ORF	RG1203	TAAGGGCGGGACAGCTAGTA	512
		RG92		
A3-2	ECH_0187 ORF	RG203	AAAATTTTGCCAGCGTAATA	364
		RG92		
A4-1(2a)	ECH_0669 ORF	RG1601	CAGCTCCTTTGGTATGAATC	458
		RG92		
A4-3	ECH_0561 ORF	RG236	TCTCTTATTGGAATGCCATC	448
		RG92		
B1-2	ECH_0655 ORF	RG277	TGATAATCAAAATGGAGTCG	411
		RG92		
B4-1	ECH_0665 ORF	attB2	ATTATATTTCTCTGATGATGACATTG	618
		RG92		
B5-1	ECH_0600 ORF	RG257	GTGATTGATTATAATAATCTGACATTTC	186
		RG92		
B6-1	ECH_1144 ORF	RG220	TTCCCAACCAAAATAATCAG	195
		RG92		
C1-1	ECH_0475 ORF	RG222	TTATGCCTAATTTTGGTGCT	402
	(aadA F1)	RRG1202	CAGTTGGAAGAATTTGTTCACTACGT	
C2-1 (2b)	ECH_0666 ORF	RG465	CTTTTATGCATGGTCCAACT	393
		RG92		
C2-3	ECH_0039 ORF	RG239	AAAAACTAACCCTGAAATTCT	236
		RG92		
C3-2	ECH_1127 ORF	RG190	ATGAGCAAAAAAAAGTTTATTACA	233
		RG92		
C4-1 (2b)	ECH_1038 ORF	RG196	AGCTCATTGCTTATTTCTCG	420
		RG92		
C6-3 (2a)	ECH_0945 ORF	RG197	ATGTGAAGGGAAGATTGTTG	447
		RG92		
D1-4	ECH_0104 ORF	RG479	ATGTCTAGTGTAGCTGCTTGTG	204
		RG92		
D3-1	ECH_0614 ORF	RG227	CATTTAATCCTGCGACATTT	315
		RG92		
D3-2	ECH_0368 ORF	RG233	CAATTGATCCAATTCTCGTT	291
		RG92		

Table S1; continued……

Mutant code	Locus	Name	Sequence (5' to 3')	Size(bp)
D3-4	ECH_0866 ORF	RG245<br>RG92	CAGCTGCAATTTTAGAACAA	370
D4-1	ECH_0242 ORF	RG228<br>RG92	ACATTATATTCCGGGAAAGG	215
D4-3	ECH_0837 ORF	RG244<br>RG92	TTCAGCTTGTGCTACACATC	315
D4-4	ECH_0666 ORF	RG250<br>RG92	TGTTCATGTGCTAATCCAGA	338
D5-4	ECH_0329 ORF	RG251<br>RG92	GGAGGAGACACTGGATACAA	428
TR31	ECH_1144 ORF	RG219<br>RG92	ATCTCGTGAGTCCACTGTTC	149
s33 C7	ECH_0525 ORF<br>(mCherry R1)	RG668<br>RG97	GAGAAGACACTCCTGCTC<br>TCCGCAGGATGTTTCACATA	599
s33 E5	ECH_0251 ORF	RG673<br>RG97	TTACCACGCTAGGAAACT	598
s33 E8	ECH_0878 ORF	RG676<br>RG97	AACTTGTGCCTCTCTAGC	1203
s34 A2	ECH_0843 ORF	RG681<br>RG97	TGATCAATTGAATTTGGA	780
s34 C8	ECH_0445 ORF	RG688<br>RG97	GAAAAATTAGGAGGCTTG	673
s34 D8	ECH_1110 ORF	RG694<br>RG97	CATCTTGCGGTACTGATA	696
s34 E3	ECH_0592 ORF	RG666<br>RG97	ACTTCCAAACCAATTTTT	604
A2-1 (4c)	ECH_0372 0373	RG457<br>RG92	GTGGCATATACTCTTCTATCTTGT	445
A2-3	ECH_0705 0706	RG455<br>RG92	ATAGGGTCTGCTGTTGAAAA	464
A3-1	ECH_0282 0283	RG417<br>RG92	ACATGGCTAATGCAGAAGAT	867
A3-3 (2b)	ECH_0699 0670	RG433<br>RG92	TTCCAAAAGAAAATCTGCAT	712
A4-2	ECH_1065 1066	RG430<br>RG92	ATACCAGAACCACTCACCTG	765
B2-3	ECH_0149 0150	RG439<br>RRG1202	CATACAAAGACAACGGGAAT	585
B3-3	ECH_1148 1149	RG444<br>RG92	GTGCTGTAATCCAAGGTGAT	2413

Table S1; continued……

Mutant code	Locus	Name	Sequence (5' to 3')	Size(bp)
B5-2	ECH_0930 0931	RG1206	TCCCAAAAACATAAGGGTCA	562
		RG92		
B6-2 (2a)	ECH_0124 0125	RG474	CAACAACAAAACCTGCATTA	514
		RRG1202		
B6-2 (2b)	ECH_1044 1045	RG1624	TCATTGAATGCTGAGATCCT	252
		RG92		
B6-3	ECH_0579 0580	RG445	CACAATACCTTGTTGCACAG	590
		RRG1202		
C6-3 (2b)	ECH_0894 0895	RG1613	TTGCTGATATTTCTTCAACA	153
		RG92		
D1-1	ECH_0605 0606	RG424	ATATTGAACCTGTTGCTGCT	585
		RRG1202		
D1-3	ECH_0083 0084	RG478	GGCCCTAGTTCTCTTACGAT	860
		RG92		
D2-1	ECH_0769 0770	RG426	TAACCATTTAGGCATCCAAG	826
		RRG1202		
D5-3	ECH_0760 0761	RG274	GCTACACGAAATGGGTAGAG	606
		RG92		
D6-3	ECH_0995 0996	RG452	GCTATTTTCATTGCAGTGGT	1179
		RG92		
s33 A7	ECH_1081 1082	RG658	TATCAAACCAGAACCACA	1082
		RG97		
s33 B7	ECH_0593 0594	RG663	TTTTAACGAACGTACCTG	1048
		RG97		
s33 E7	ECH_0750 0751	RG672	TGGATCAGTTGCAAGATA	1042
		RG97		
s33 F3	ECH_0579 0580	RG679	ATGATTGATTTATATAATATGAAATTAC	732
		RG97		
s34 B1	ECH_0657 0658	RG687	CGGAGAGAGAGGGATT	753
		RG97		
s34 D7 7/31	ECH_0537 0538	RG690	CATTAATGCGTAATGCTC	971
		RG97		
s34 E1	ECH_1008 1009	RG696	AATTGCAGGTTTAATTCC	972
		RG97		
Primers for RT-PCR analysis of mutations within the gene ORFs:				
A1-1	ECH_0113 ORF	RG254	CCGTTATCACATCAACTCCT	350
		RG256	CCAACCTTAACCTTTTCAAC	
A3-2	ECH_0187 ORF	RG203	AAAATTTTGCCAGCGTAATA	367
		RG204	TGCAGAGATGTTGTGTGTTT	
A4-3	ECH_0561 ORF	RG236	TCTCTTATTGGAATGCCATC	419
		RG237	CCACCTGAATTTTTCCAAT	

Table S1; continued……

Mutant code	Locus	Name	Sequence (5' to 3')	Size(bp)
B1-2	ECH_0655 ORF	RG277	TGATAATCAAAATGGAGTCG	322
		RG279	TTAACTATTGATATTACAATGACC	
B5-1	ECH_0600 ORF	RG257	GTGATTGATTATAATAATCTGACATTTC	144
		RG259	TCAAGTTAATGCAAGCACTG	
B6-1	ECH_1144 ORF	RG220	TTCCCAACCAAAATAATCAG	382
		RG219	ATCTCGTGAGTCCACTGTTC	
C1-1	ECH_0475 ORF	RG222	TTATGCCTAATTTTGGTGCT	287
		RG224	CATATTCATGAAGTCTGTATTTTT	
C2-1 (2b)	ECH_0666 ORF	RG465	CTTTTATGCATGGTCCAACT	310
		RG467	ATCACAAAAGGTGGCATAAT	
C2-3	ECH_0039 ORF	RG239	AAAAACTAACCCTGAAATTCT	286
		RG241	TCCAGGTTTTGACATAGGAC	
C3-2	ECH_1127 ORF	RG190	ATGAGCAAAAAAAAGTTTATTACA	588
		RG192	ATCTTCACCAATACCTGCAC	
C4-1 (2b)	ECH_1038 ORF	RG195	TTGCAGCAAGATGCTAATAA	413
		RG196	AGCTCATTGCTTATTTCTCG	
C6-3 (2a)	ECH_0945 ORF	RG199	TCCATTGCTAAGGGTAATTT	298
		RG200	TGCATATGACTACCCTGATCT	
D1-4	ECH_0104 ORF	RG479	ATGTCTAGTGTAGCTGCTTGTG	125
		RG480	GCATATTACTTCATCATACAACG	
D3-1	ECH_0614 ORF	RG225	CATAGGCAATACCGAAGATG	422
		RG227	CATTTAATCCTGCGACATTT	
D3-2	ECH_0368 ORF	RG233	CAATTGATCCAATTCTCGTT	317
		RG235	GATTTTCCTGAACCAATGAA	
D3-4	ECH_0866 ORF	RG245	CAGCTGCAATTTTAGAACAA	435
		RG247	CGTTCCTTACTGGTTTTTCA	
D4-1	ECH_0242 ORF	RG228	ACATTATATTCCGGGAAAGG	146
		RG229	TTGCCTAAATGAATCTGACA	
D4-3	ECH_0837 ORF	RG242	ATGCAAGGTTTATACATCAAGTC	255
		RG244	TTCAGCTTGTGCTACACATC	
D4-4	ECH_0666 ORF	RG248	ATGTATGCGGATTTTTGTAC	269
		RG250	TGTTCATGTGCTAATCCAGA	
D5-4	ECH_0329 ORF	RG251	GGAGGAGACACTGGATACAA	348
		RG253	TTACATAGCAAAATTTTTTACAAATTC	
s33 C7	ECH_0525 ORF	RG667	TTGTCTAAGCAGCAAGAA	127
		RG668	GAGAAGACACTCCTGCTC	
s33 E5	ECH_0251 ORF	RG673	TTACCACGCTAGGAAACT	147
		RG674	CATTAGAATGCGTCGTAG	
s33 E8	ECH_0878 ORF	RG675	ACGTTGTAAATCAAGTGC	1051
		RG676	AACTTGTGCCTCTCTAGC	

Table S1; continued……

Mutant code	Locus	Name	Sequence (5' to 3')	Size(bp)
s34 A2	ECH_0843 ORF	RG681	TGATCAATTGAATTTGGA	306
		RG683	AAAACACTTCCAGTGATG	
s34 C8	ECH_0445 ORF	RG688	GAAAAATTAGGAGGCTTG	153
		RG689	GGAAACTAGGGTTTTTGA	
s34 D8	ECH_1110 ORF	RG694	CATCTTGCGGTACTGATA	232
		RG695	TTAGGAGCATGTGGATAA	
s34 E3	ECH_0592 ORF	RG665	AGCGGAATTTCTATTGTT	102
		RG666	ACTTCCAAACCAATTTTT	
Primers for RT-PCR analysis of genes up and downstream of intergenic insertions:				
A3-1	ECH_0282	RG417	ACATGGCTAATGCAGAAGAT	259
		RG418	ATCTGTCCATGCTACTTGCT	
	ECH_0283	RG419	AGGCATAGTAACATCACACAAA	106
		RG420	TGCTAATTATAGCATAAAAAGGTT	
D1-1	ECH_0605	RG421	GGAATCCTCCTAAATTTGCT	169
		RG422	TCATCATCTTCATGACTCCA	
	ECH_0606	RG423	TCAAAATCAGTTATCATCTGG	106
		RG424	ATATTGAACCTGTTGCTGCT	
D2-1	ECH_0769	RG425	CGTGGTTTTGGAGTATTGAT	156
		RG426	TAACCATTTAGGCATCCAAG	
	ECH_0770	RG427	TTGATTAAAATTATTAAGCAT	114
		RG428	TTAAAAATTAATTGCATTATAC	
A2-3	ECH_0705	RG453	TTTCCGAATGTACGTAAGGT	162
		RG454	AGTCTGTGAATGCCACTTTC	
	ECH_0706	RG455	ATAGGGTCTGCTGTTGAAAA	133
		RG456	TCTTTGGCTTAGCAACTTGT	
B2-3	ECH_0149	RG437	ATAACAGCGTTTTCCCTACA	220
		RG438	ATTCCATTATCAATGCTGCT	
	ECH_0150	RG439	CATACAAAGACAACGGGAAT	237
		RG440	ACATAACACCGAAGAGGAAA	
B3-3	ECH_1148	RG441	AAAGTGACGATGCTGCTAAT	231
		RG442	CTTCTGCTGGTACAGCTTCT	
	ECH_1149	RG443	AGTTGTTTTCGACCAAAGAA	192
		RG444	GTGCTGTAATCCAAGGTGAT	
B6-3	ECH_0579	RG445	CACAATACCTTGTTGCACAG	272
		RG446	ACCATTTATCGCAATTTGTT	
	ECH_0580	RG447	GCAAGAATTATGGGAACAAA	66
		RG448	TGTATTTTTGTATTATTTTGTGATG	

Table S1; continued……

Mutant code	Locus	Name	Sequence (5' to 3')	Size ((bp)
D6-3	ECH_0995	RG449	GGATAAACACATCCTCAACG	108
		RG450	GTCGCCATATACACAAGGTT	
	ECH_0996	RG451	ATAATATCAGGCAACGGAGA	167
		RG452	GCTATTTTCATTGCAGTGGT	
Primers for nested PCRs used for the animal experiments:				
Gene specific primer at mutations		listed above		
Amtr-specific first primer		RRG1258	ATGAAGGAGGGCCCTTATGG	
Amtr-specific nested primer		RG92		
aadA-specific first primer		RRG1200	GTTACGGTGACCGTAAGGCTTG	
aadA-specific nested primer		RRG1202		
mCherry-specific first primer		RG97		
mCherry-specific nested primer		RRG1257	AAACAAATACCTTTAACATCATTAAACCATTTC	
